# Protocol for modulating the noradrenergic pathway from locus coeruleus to heart to prevent sudden unexpected death in epilepsy in mouse models

**DOI:** 10.1016/j.xpro.2023.102403

**Published:** 2023-06-30

**Authors:** Qing Xu, YuLing Wang, XiTing Lian, Lu Liu, WeiHui Shao, JiaXuan Gu, LeYuan Gu, Qian Yu, YuanLi Zhang, ZhuoYue Zhang, HaiXiang Ma, Yue Shen, HongHai Zhang

**Affiliations:** 1 Department of Anesthesiology, Affiliated Hangzhou First People’s Hospital, Zhejiang University School of Medicine, Hangzhou 310006, China; 2 Department of Anesthesiology, the Fourth Clinical School of Medicine, Zhejiang Chinese Medical University, Hangzhou 310006, China; 3 Westlake Laboratory of Life Sciences and Biomedicine, Hangzhou 310006, China

**Keywords:** Neuroscience, Cognitive Neuroscience, Behavior

## Abstract

The locus coeruleus (LC) and noradrenergic neurotransmission are involved in the regulation of sudden unexpected death in epilepsy (SUDEP). Here, we present a protocol for modulating the noradrenergic pathway from LC to heart to prevent SUDEP in acoustic and pentylenetetrazole-induced DBA/1 mouse models of SUDEP. We describe steps for constructing SUDEP models, calcium signal recording, and electrocardiogram monitoring. We then detail measurement of tyrosine hydroxylase content and activity, β1 and p-β1-AR content, and destruction of LC^NE^ neurons.

For complete details on the use and execution of this protocol, please refer to Lian et al.^1^

## Highlights

• A detailed protocol for viral infusion and implantation of optical fibers into the LC
• Immunofluorescence staining scheme for heart and brain tissues
• The measurement of the activity of TH and β1-AR in the heart and peripheral blood
• ECG recording of both acoustic and PTZ-induced DBA/1 mouse models of SUDEP

## Abstract (editor highlights)

Publisher’s note: Undertaking any experimental protocol requires adherence to local institutional guidelines for laboratory safety and ethics.

## Graphical abstract

**Figure:**
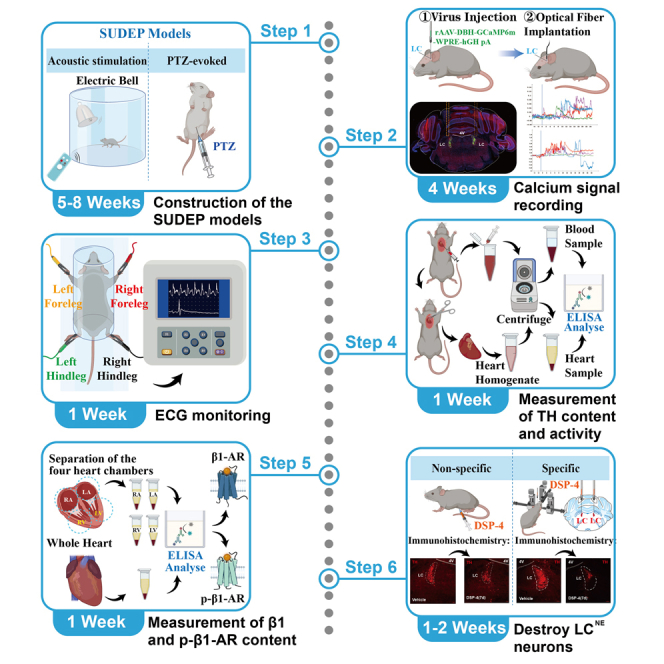

## Before you begin

In the DBA/1 mouse model, both cardiac and respiratory functions are suppressed simultaneously during the induction of S-IRA and SUDEP, suggesting that the autonomic nervous system, including the sympathetic and parasympathetic nervous systems, is involved in S-IRA and SUDEP.^2^ Improving the efficiency of synaptic transmission of NE in the brain has been shown to effectively reduce the incidence of SUDEP.^3^^,^^4^ Thus, we hypothesize that the interaction between noradrenergic neurons in the LC and β1-AR on cardiomyocytes plays a key role in the regulation of SUDEP, which could be a potential target for SUDEP prevention and provide a new perspective for elucidating the pathogenesis of SUDEP. The approach is mainly based on calcium signaling, ECG recordings and ELISA (Enzyme-Linked Immunosorbent Assay) to illustrate the role of the brain-heart axis in the occurrence of SUDEP.

### Institutional permissions

All experimental procedures are in line with the National Institutes of Health Guidelines for the Care and Use of Laboratory Animals, approved by the Animal Advisory Committee of Zhejiang University, and followed proper animal use protocols and institutional guidelines. The users of the protocol must obtain similar permissions from corresponding institutions.

### Breeding animals

**Timing: 4–5 months prior to the experiment**

This section describes the process of breeding the mouse model of S-IRA induced by acoustic stimulation.

1. House and breed DBA/1 mice in the Animal Center of Zhejiang University School of Medicine and give them rodent food and water ad libitum in the soundproof room to protect the sensitivity to sound.^5^
2. Use DBA/1 mice of either gender in the experiments.^5^ Use both male and female DBA/1 mice in the experiment according to the reproduction.

### Seizure induction and resuscitation

This section describes the ways of inducing seizures in the two models and the rescue measures after the occurrence of S-IRA.

3. For the acoustic stimulation model, each DBA/1 mouse in 26–28 days is placed in a cylindrical plexiglass chamber in a sound-isolated room, and generalized audiogenic seizures (AGSz) are evoked by an electric bell (96 dB SPL, Zhejiang People’s Electronics, China). For detailed information please refer to Wang et al.^6^

***Note:*** DBA/1 mice were “primed” starting from postnatal days 26–28 by subjecting to acoustic stimulation daily for 3–4 days to establish consistent susceptibility to audiogenic seizures and S-IRA. Use the primed DBA/1 mice to conduct experiments. The unprimed DBA/1 mice that did not have seizures as well as S-IRA within 3–4 days of acoustic stimulation, were not included in the experiments. Acoustic stimulation was given for a maximum duration of 60 s or until the onset of tonic seizures and S-IRA in most mice in each group. It normally takes 10–20 seconds to induce seizures of the DBA/1 mouse. S-IRA usually occurs 3S after seizure onset. When the DBA/1 mouse develops respiratory arrest after seizures, we record the state as S-IRA. Resuscitate DBA/1 mice with S-IRA within 5 s after the final respiratory arrest using a rodent respirator (180 strokes/minute, 1:1.5 of I/E and a volume of 1 cc with room air).^6^ Confirm the susceptibility of primed DBA/1 mice to SIRA before 24 h of drug or vehicle treatment.

4. For the pentylenetetrazole (PTZ)-evoked seizure model, evoke S-IRA in all non-primed DBA/1 mice by a single intraperitoneal inject (IP) dose of PTZ at 75 mg/kg. Observe the mice for 1 h after PTZ injection.^4^

***Note:*** Formal experiments are performed in mice at 8 weeks of age (10–15 g).

### Reagents preparation

**Timing: 40 min**

This section describes the preparation and storage methods of reagents required for the experiment.

5. For anesthetization: dissolve 3.5 g chloral hydrate in 100 mL sterilized 0.9% saline to make 3.5% chloral hydrate solution.

***Note:*** Store the chloral hydrate (3.5%) at room temperature (RT, 15°C–25°C) for up to 1 month.

6. According to the corresponding drug concentration, dissolve atomoxetine, esmolol and N-(2-chloroethyl)-N-ethyl-2-bromobenzylamin hydrochloride (DSP-4) in 0.9% saline.

***Note:*** Compound the drug when it is used, avoid storage for more than 3 h and do not leave it at 21°C–26°C for too long.

7. Take out rAAV-DBH-GCaMP6m-WPRE-hGH pA virus, and put it on the ice immediately before viral loading and wrap it in tinfoil to protect it from light.

### Preparation before stereotactic surgery

**Timing: 1 h**

This section describes the environment and equipment preparation of mice before stereotactic surgery.

**Figure 1:**
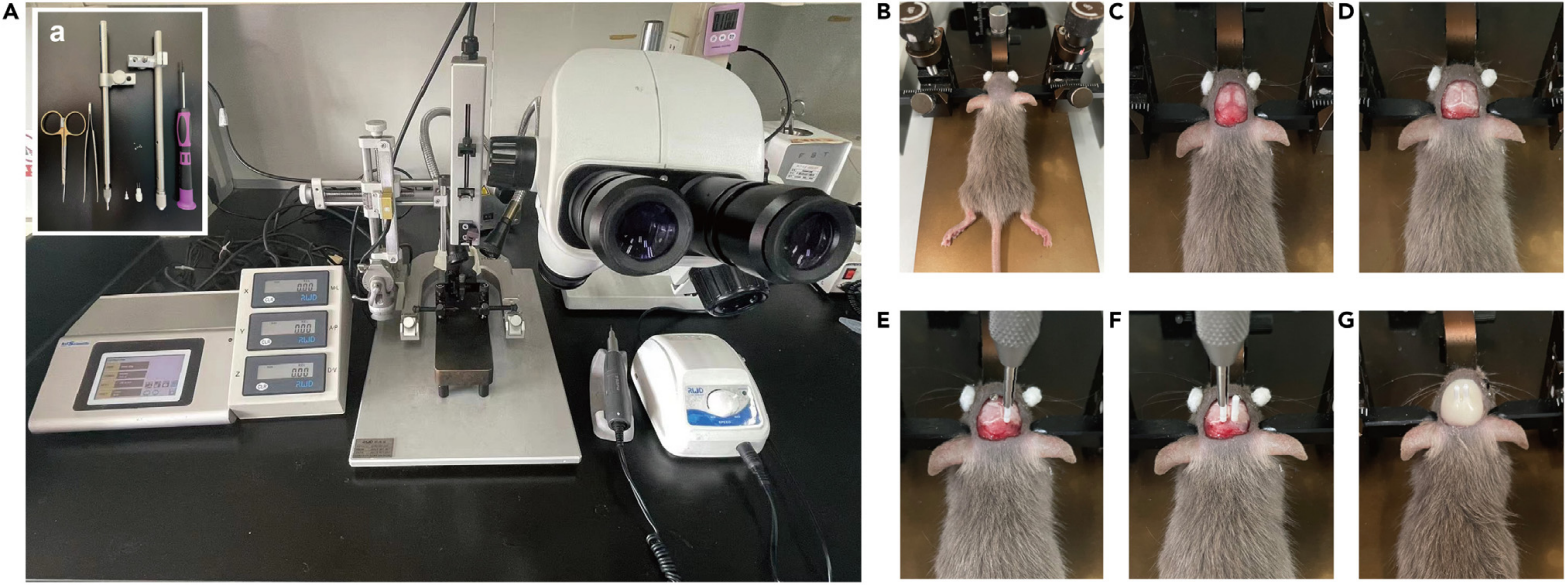
Stereotactic surgical materials and key procedures (A) Surgical tool for optical fiber implantation and stereotaxic instrument. (B) Fix the head of mice on the stereotaxic device by ear bars. Then hang the front tooth on the tooth bar. Smear erythromycin eye ointment on the surface of mouse eyeballs and then cover them with sterile cotton balls. (C) Remove the skin of the mouse head, and wipe the connective tissue on the skull surface with a cotton ball soaked in alcohol. (D) Wipe the bone seam with a cotton ball dipped in hydrogen peroxide to expose bregma and lambda. (E) According to the parameters of the LC, carefully sand the skull with a dental drill and implant the optical fiber. (F) The LC on the other side is also implanted with an optical fiber. (G) Use dental cement to fix the optical fiber after the operation. LC, locus coeruleus.

***Note:*** Before virus loading, hang the gauge needle and observe if there is a droplet on the tip of the needle to test the air impermeability.

8. Connect the gauge needle (10 μL, virus injection) to the microinfusion pump, and assemble the stereotaxic instrument and dental drill (Figure 1A).

9. Turn on a heating pad to keep the body temperature (37°C) of anesthetized mice.
10. Intraperitoneal injection of 3.5% chloral hydrate solution into the mouse waiting for 5–10 min, check the mouse’s responsiveness to noxious stimuli.

***Note:*** Weigh the mice and calculate the dosage of chloral hydrate solution used for anesthetization. In this protocol, for 15 g male DBA/1 mice, 8 weeks old, 0.23 mL of 3.5% chloral hydrate for adult DBA/1 mice, 15 mL/kg body weight is recommended. Compared with C57BL/6J mice, DBA/1 mice are tolerant to anesthetics. After 10 min, if the anesthetic effect is not ideal, intraperitoneally inject 0.05 mL of 3.5% chloral hydrate.

## Key resources table

**Table:** 

REAGENT or RESOURCE	SOURCE	IDENTIFIER
**Antibodies**		

Rabbit anti-TH	Merck-Millipore	AB152(1:1000)
Donkey anti-rabbit Alexa 546	Thermo Fisher Scientific	A10040(1:1000)
Rabbit anti-β1-AR	Alomone	AAR-023(1:400)

**Bacterial and virus strains**		

rAAV-DBH-GCaMP6m-WPRE-hGH pA	Brain VTA Technology Co., Ltd.	N/A

**Chemicals, peptides, and recombinant proteins**		

PTZ	Sigma-Aldrich, St. Louis, MO	Cat #P6500
Atomoxetine	Sigma-Aldrich, St. Louis, MO	Ca #Y0001586
Esmolol	Qilu Pharmaceutical Co., Ltd.	H19991058
DSP-4	Sigma-Aldrich, St. Louis, MO	C8417
Chloral hydrate	Sangon Biotech (Shanghai) Co., Ltd. Shanghai, China	I418BA0004
Enzyme-linked immunosorbent assay (ELISA) kit	Yan Sheng Biological Technology Co., Ltd.	YS-M195
DAPI	Beyotime Biotechnology, Shanghai, China	Cat #C1002
PBS	Beijing Solarbio Science & Technology Co., Ltd.	Cat #P1010
PFA	Sinopharm Chemical Reagent Co., Ltd	30525-89-4
Mounting medium	Beijing Solarbio Science & Technology Co., Ltd.	Cat #S2100
Triton X-100	Sigma-Aldrich, St. Louis, MO	T9284
BSA	BioFroXX, Germany	9048-46-8
NDS	Tianhang Biotechnology Co., Ltd, Zhejiang, China	80230–6412
Saline	China Otsuka Pharmaceutical Co., Ltd. Tianjin, China	DB01-1-0701

**Experimental models: Organisms/strains**		

DBA/1 mice	The Animal Center of Zhejiang University School of Medicine, Harlan Laboratories	Male/Female, 8 weeks old

**Software and algorithms**		

SPSS	Chicago, IL, USA	http://www.spss.com.cn

**Others**		

Rodent respirator	Holliston, MA, U.S.A.	Harvard Apparatus 680
Glass electrode	Sutter Instrument, Novato, USA	B100-58-10
Microinfusion pump	Kd Scientific	N/A
Microliter syringes	Shanghai Gaoge Industry and Trade Co., Ltd, China	G019105
Stereotaxic device	RWD Life Science Inc., Shenzhen, China	68,018
Dental drill	RWD Life Science Inc., Shenzhen, China	78001
Denture base materials	Shanghai New Century Dental Material Co., Ltd, China	N/A
Artificial teeth resin	Shanghai New Century Dental Material Co., Ltd, China	N/A
Glue	Komax, Zhejiang, China	N/A
The mouse atlas of Paxinos and Franklin	4th Edition, 2013	N/A
Syringes	JIANGXI HONGDA MEDICAL EQUIPMENT GROUP LTD.	N/A
Medical cotton balls	JIANGXI HONGDA MEDICAL EQUIPMENT GROUP LTD.	N/A
Photometry system and laser	Inper, Hangzhou, China	N/A
Thermo Enzyme Labeling Instrument	Thermo Fisher Scientific	5250040
Optical fiber	Inper, Hangzhou, China	FOC-W-1.25-200-0.37-4.0
LC guide cannulas	RWD Life Science Inc., China	62070

## Materials and equipment

**Table:** Sealing fluid

Reagent	Final concentration	Amount
NDS	100%	100 μL
BSA	10%	100 μL
Triton X-100	20%	15 μL
1× PBS	N/A	785 μL
**Total**	**N/A**	**1 mL**

Note on storage conditions: 4°C, maximum time for storage: 1 day.

**Table:** First antibody diluent

Reagent	Final concentration	Amount
NDS	100%	10 μL
BSA	10%	100 μL
Triton X-100	20%	15 μL
1× PBS	N/A	875 μL
**Total**	**N/A**	**1 mL**

Note on storage conditions: 4°C, maximum time for storage: 1 day.

**Table:** Second antibody diluent

Reagent	Final concentration	Amount
NDS	100%	10 μL
1× PBS	N/A	990 μL
**Total**	**N/A**	**1 mL**

Note on storage conditions: 4°C, maximum time for storage: 1 day.

Note:

• 10% BSA solution: add 10 g BSA in 100 mL 1× PBS.

Note on storage conditions: −20°C, maximum time for storage: 1 month.

• 20% Triton X-100 solution: add 2 mL Triton X-100 in 10 mL ddH_2_O.

Note on storage conditions: 4°C, maximum time for storage: 1 month.

**Table:** 3.5% Chloral hydrate solution

Reagent	Final concentration	Amount
Chloral hydrate	3.5%	0.35 g
Saline	0.9%	10 mL
**Total**	**N/A**	**10 mL**

Note on storage conditions: ambient storage, maximum time for storage: 1 month.

***Alternatives:*** Dissolve 1.0 g pentobarbital in 100 mL sterilized 0.9% saline to make 1% pentobarbital solution, for anesthetization. For adult C57BL/6J mice, 5–6 mL/kg body weight is recommended, e.g., 0.115 mL 1% pentobarbital for a 20 g mice, aged 8 weeks.

## Step-by-step method details

### Stereotactic surgery

**Timing: 3 h**

1. Shave the mouse head based on the location of the target nucleus. With reference to the AP value (−5.45 mm) of LC, our shaving range extends to the binaural level (Figure 1).
2. Fix the head of mice on the stereotaxic device by ear bars. After fixing, the head does not move forward or back, left or right, or up or down.
3. To avoid irreversible damage to the mice’s vision caused by strong light, smear eye ointment on the surface of mouse eyeballs and then cover them with sterile cotton balls (Figure 1).
4. Cut the skin of the mouse head with ophthalmic scissors, and press the saline cotton ball to stop the bleeding.
5. To expose bregma and lambda, gently wipe the bone suture with a cotton ball damped with hydrogen peroxide (Figure 1).
6. Insert four miniature screws far away bilateral LC laterally along the front and back of the skull, forming roughly a square.

***Note:*** The implantation position should be away from the bregma and target nucleus. The implant depth is 1/2 to 2/3 of the screw.

7. Gently touch bregma and lambda with the glass electrode.
  a. Level back and forth according to the DV value (difference less than 0.03 mm), then return to bregma level for horizontal leveling.
  b. After leveling left and right, double check the bregma and lambda levels.
8. According to the parameters of the injected brain region, carefully sand the skull with a dental drill at the injection site (AP-5.45 mm, ML ±0.9 mm), thinning it slowly.

***Note:*** Twist sterile cotton balls into thin threads and hook out he bone fragments at the injection site.

9. Slowly insert the glass electrode until it reaches the target nucleus.
10. Based on the mouse atlas of Paxinos and Franklin (4th Edition, 2013), microinject (100 nL, at a rate of 40 nL/min) rAAV-DBH-GCaMP6m-WPRE-hGH pA virus by microinfusion pump(Kd Scientific) according to the following stereotaxic coordinates of LC (AP-5.45 mm, ML ±0.9 mm, DV −3.65 mm).

***Note:*** It is recommended that the injection speed should be set at 10–40 nL/min. If the injection position is deep or the needle is easily blocked, the injection speed can be accelerated up to a maximum of 80 nL/min.

**CRITICAL:** During microinjection, if the tip of the needle is smooth, the dropping liquid level of the virus and liquid paraffin in the glass electrode can be observed through the microscope. The front end of the glass electrode can be trimmed with tissue scissors, and the length of the front end refers to the DV value of the nucleus. If the needle tip is too thin, it will easily cause needle blockage.

11. Do not pull out the syringe until 10 min after the injection to allow the virus to spread.
12. At the same coordinates, implant the optical fiber (FOC-W-1.25-200-0.37-4.0, Inper, Hangzhou, China) above LC for 0.10 mm (AP-5.45 mm, ML ±0.9 mm, DV −3.65 mm ) (Figure 1).
13. Use dental cement to fix optical fibers.

***Note:*** The time for bone cement to set completely is approximately 10 min. After the surgery, place the mice on a heating pad. 0.5–1 h after surgery, the mice will awaken. To avoid the fiber optic on the mouse's head getting stuck in the feeding trough, sprinkle the feed on the bedding.

***Note:*** When dental cement is used to secure the sleeve, the cement must completely cover the screw and only the upper part of the guide sleeve is visible outside the implant structure. In order to avoid adverse effects on the recovery and physiological activities of mice, excessive dental cement should not be used.

***Note:*** After implantation, let the virus express for 3 weeks.

### Immunohistochemistry

14. Inject 3.5% chloral hydrate intraperitoneally to anesthetize mice. Other anesthesia methods are acceptable.

***Note:*** Recommended dosage of anesthetic is 15 mg/kg of 3.5% chloral hydrate.

**Figure 2:**
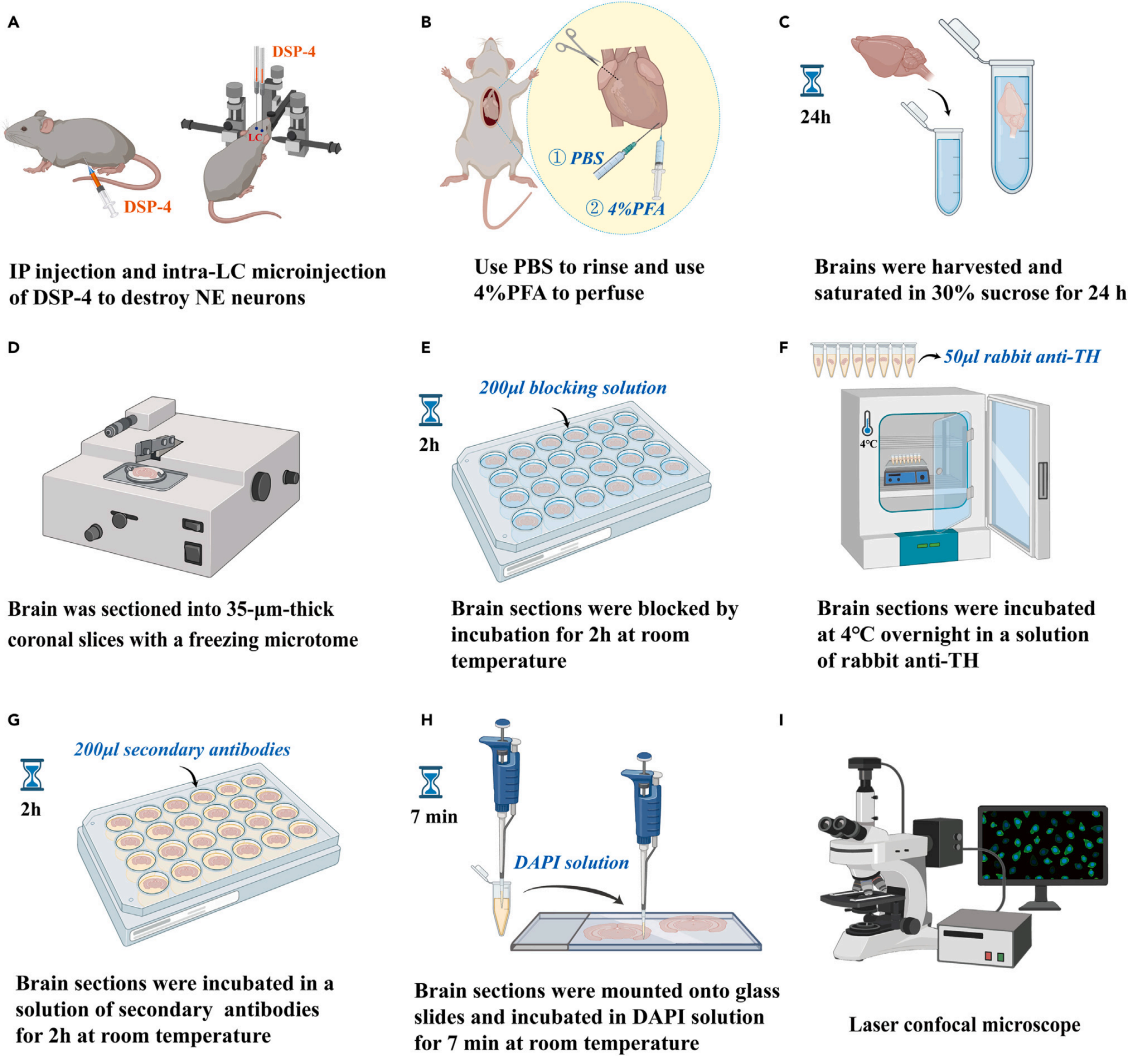
Key immunohistochemical procedures for testing DSP-4 disruption of LC^NE^ neurons (A) Two methods for injecting DSP-4 to destroy the LC^NE^ neurons: IP injection of DSP-4 for non-specific destroying of LC^NE^ neurons; Intra-LC injection of DSP-4 for specific destroying of LC^NE^ neurons. (B) Perfuse the brain with cold PBS to rinse the blood, and then perfuse the brain with 4% PFA. (C) Strip the brain carefully and saturate it in a 30% sucrose solution for 24 h. (D) Slice the brain into 35-μm-thick coronal slices with a freezing microtome. (E) Wash the brain slices with PBS, and then block them by incubation for 2 h at room temperature in the blocking solution (10% normal donkey serum, 1% bovine serum albumin, and 0.3% Triton X-100 in PBS). (F) The brain slices are incubated at 4°C overnight in a solution of rabbit anti-TH. (G) The brain slices are incubated in a solution of secondary antibodies for 2 h. (H) The brain slices are mounted onto glass slides and incubated in DAPI solution for 7 min. (I) Immunohistochemically stained slices were photographed using an Olympus microscope (VS.120) and a laser confocal microscope (Nikon A1). IP, intraperitoneal; PFA, paraformaldehyde; PBS, phosphate-buffered saline; LC, locus coeruleus.
16. Soak brain tissue in 4% PFA and post-fixation time is 24 h.
17. Strip the brain carefully and saturate it in a 30% sucrose solution for 24 h.

***Note:*** When extracting the heart, take care that the injection speed of the syringe. It is highly recommended to use infusion pump to avoid the deformation of the heart.

15. Perfuse the brain with cold PBS to rinse the blood from the left ventricle, and then perfuse with 4% PFA (Figure 2).

18. Slice the brain into 35-μm-thick coronal slices with a freezing microtome.

***Note:*** Slice the heart into 15-μm-thick coronal slices with a freezing microtome.

19. Wash the brain slices.
  a. Place the brain slices in the hole plate, fill each hole with 1× PBS from the side wall.
  b. Place them on a shaker (40–50 r/min) for 5 min.
  c. After 5 min, absorb the waste liquid, and wash 3 times (Figure 2).
20. Seal the brain slices.
  a. Prepare sealing fluid on the ice box (1 mL contains 100 μL 100% NDS, 100 μL 10% BSA, 15 μL 20% triton and 785 μL 1× PBS).
  b. Add 200 μL sealing fluid to each hole.
  c. Place the plate on a shaker (10 r/min) at room temperature for 2.5 h.

***Note:*** After thawing the NDS BSA, shake the solution evenly before use. Compound sealing fluid, first antibody diluent and second antibody diluent when it is used.

21. Prepare the first antibody diluent (1 mL contains 10 μL 100% NDS, 100 μL 10% BSA, 15 μL 20% triton and 875 μL 1× PBS) on an ice box, add TH antibody (1:1000), and shake evenly.
22. Add the first antibody to an octuple tube (50 μL/well), place it on a shaker (10 r/min) in 4°C refrigerator, and incubate it overnight
23. The next day, after warming the sample to room temperature (RT, 15°C–25°C), place the sample in a hole plate filled with PBS, place it on a shaker (40–50 r/min), and wash 10 min. Repeat for 3 times.
24. Prepare the second antibody diluent (1 mL contains 10 μL 100% NDS and 990 μL 1× PBS) on the ice box, add the second antibody and store it away from light.

***Note:*** Subsequent steps are light avoidance operation.

25. Add 200 μL of secondary antibody to each well and incubate it in a shaker (10 r/min) in dark for 1 h.
26. After incubation, remove the secondary antibody and add 1× PBS, place it on a shaker (40–50 r/min) for 15 min and wash 3 times.
27. Use a line marker to gently fix the brain slice in the orifice plate onto the slide, fully unfold the brain slice, and avoid bending corners.
28. After the brain slices are completely dry, drop the DAPI diluted with PBS (1:4000) onto the slide, and completely covers all the brain slices. Incubate in dark for 7 min.

***Note:*** Completely cover the brain slices with DAPI. Incubate in dark for 7 min.

29. Discard the DAPI, drop the 1× PBS onto the slides, and wash 7–10 times.
30. After the brain slices dry thoroughly, add 2–3 drops of sealing agent, cover with an appropriate cover glass, and store in dark for 4°C before imaging.

### Fiber photometry experiment

**Timing: 2 h**

31. Animal handling and habituation: place the mice into the experimental environment 10 min in advance.
32. Connect the photometry recording system to the experimental animals through ceramic sleeves, optical fibers, rotary joints and optical fiber jumpers (Figure 6).

***Note:*** Since calcium signal recording is susceptible to external light, we recommend that it be performed in a dark environment.

33. Set the required experimental parameters on the device, such as pulse width, frequency and duration.

***Note:*** In our experiment, the fiber photometry system (Inper, Hangzhou, China, C11946) uses a 488 nm diode laser. The parameter for pulse width, frequency and duration: 20 Hz, 20-ms pulse width, 15 mW and 60 min.

34. In the fiber photometry experiment, a 410 nm signal needs to be recorded simultaneously.

***Note:*** The 410 nm laser does not affect the fluorescence protein signal, but it can reflect the signal changes of the above interference factors to reflect the background noise signal, and can re-eliminate the background noise signal to directly obtain the real calcium signal data.

35. After the animals acclimate to the environment, start recording.

### ELISA sampling

**Timing: 120 min**

**Figure 3:**
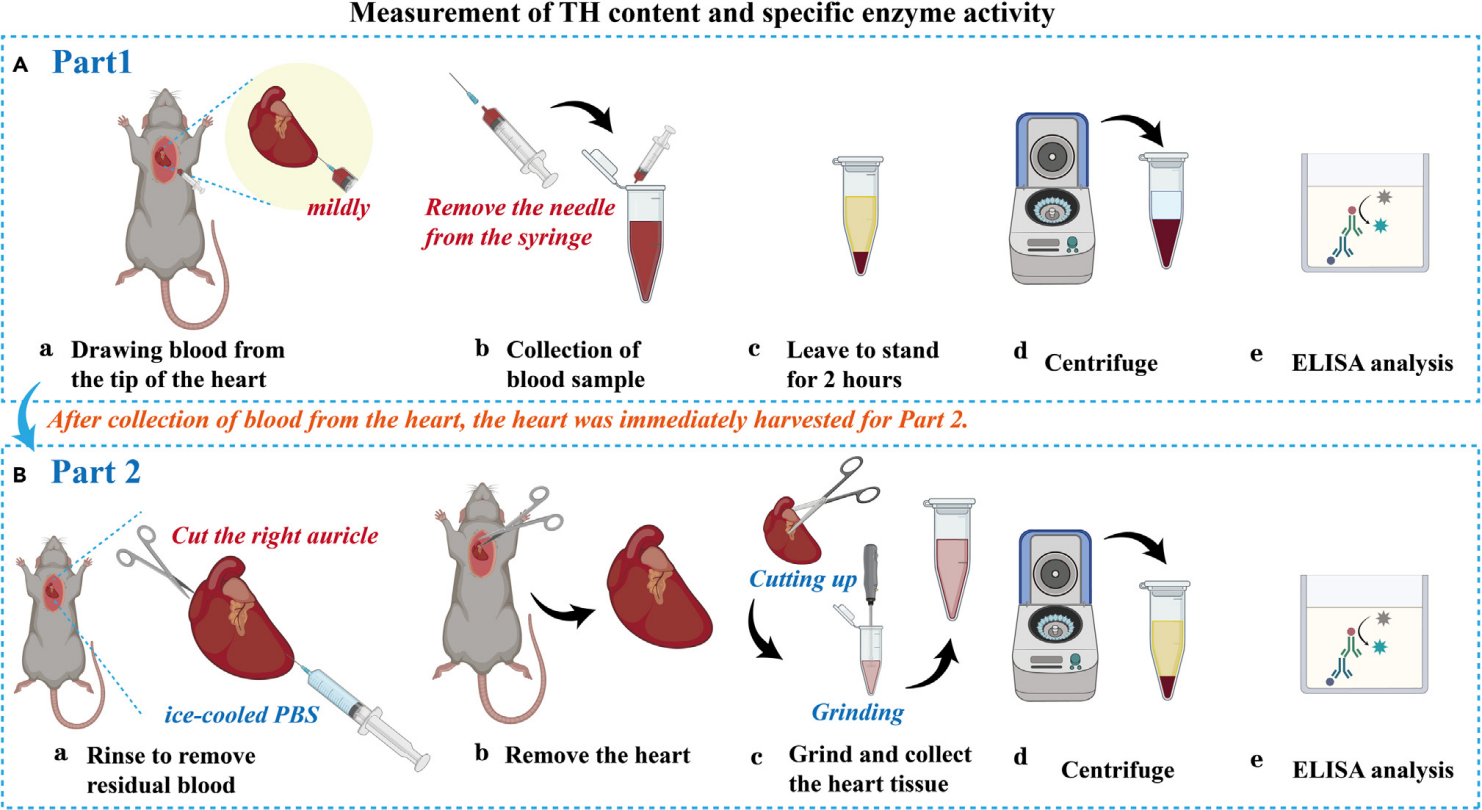
Key procedures for the measurement of TH content and specific activity in the whole heart and heart blood (A) Plasma sample preparation: a. Inject the needle (the needle was fully moistened with heparin) from the apex of the heart and draw blood as mildly as possible. b. When transferring blood from a syringe to a centrifuge tube, remove the needle from the syringe and slowly push the syringe to drip the blood into the tube. c. The collected blood samples are left to stand at room temperature for 2 h d. After standing for 2 h, centrifuge the samples for 15 min (1000 rpm) to obtain plasma. e. The TH content and specific enzyme activity of plasma from the heart are measured using an ELISA kit. (B) Heart tissue sample Preparation: a. After blood collection, perfuse the heart with cold PBS immediately. b. Remove the heart quickly. c. Use ophthalmic scissors to crush the heart tissue, then whip the solution with an ultrasonic knife until the solution is uniform. d. Pre-cool the centrifuge to 4°C and centrifuge the samples for 20 min (12000 rpm). e. The TH content and specific enzyme activity of heart tissue are measured using an ELISA kit. ELISA, enzyme-linked immunosorbent assay; PBS, phosphate-buffered saline.

***Note:*** It is suggested to divide the plasma into multiple parts before storage. Blood samples can be stored for 24–48 h at 2°C–8°C, 1 month at −20°C, 6 months at −70°C. The sample cannot be repeatedly frozen and dissolved, otherwise the potency will quickly decrease. It is recommended to use fresh samples for testing and not store them for too long.

**Figure 4:**
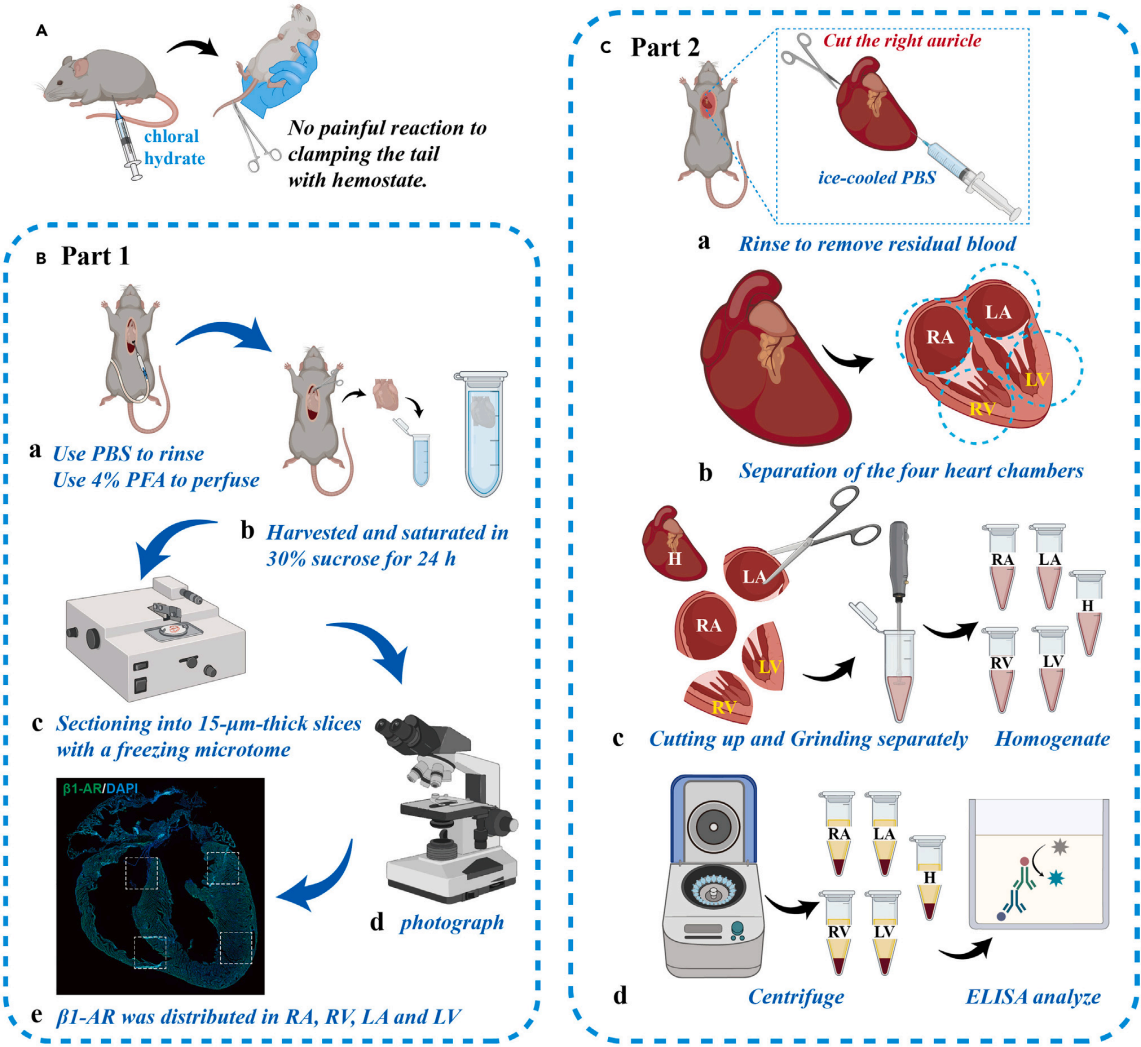
Key procedures of verifying the distribution of β1-AR and the content of β1-AR and p-β1-AR in each heart chamber (A) 3.5% chloral hydrate intraperitoneally injected to anesthetize mice (for 20 g male DBA/1 mice, 8 weeks old, 0.30 mL of 3.5% chloral hydrate, 15 mL/kg body weight is recommended). (B) Key procedures for verifying the distribution and expression of β1-AR in RA, RV, LA, and LV: a. Perfuse the heart with cold PBS to rinse the residual blood, and then perfuse the heart with 4% PFA. b. Harvest the heart and saturate it in a 30% sucrose solution for 24 h c. Slice the heart into 15-μm-thick coronal slices with a freezing microtome. d. Immunohistochemically stained slices were photographed using an Olympus microscope and a laser confocal microscope. e. Representative image shows that β1-AR is preferentially distributed in RA, RV, LA, and LV. (C) Key procedures for measuring the content of β1-AR and p-β1-AR in H, RA, RV, LA, and LV: a. Perfuse the heart with cold PBS to rinse the residual blood. b. The heart of a mouse is carefully divided into four parts using a scalpel: RA, RV, LA, and LV. c. Use ophthalmic scissors to crush the heart tissue (H, RA, RV, LA, and LV) separately, then whip the solution with an ultrasonic knife until the solution is uniform. d. The obtained tissue homogenates from H, RA, RV, LA, and LV are centrifuged for 20 min (12000 rpm) to get supernatant. The supernatant is used for the measurement of the content of β1-AR and p-β1-AR. PBS, phosphate-buffered saline; PFA, paraformaldehyde; RA: the right atrium. RV: the right ventricle. LA: the left atrium. LV: the right ventricle. H: the whole heart. β1-AR: Beta receptor1. p-β1-AR: phospho-Beta receptor1.

36. Anesthesia or intraperitoneal injection of PTZ before sampling.
  a. The control group was anesthetized by 3.5% chloral hydrate (for 20 g male DBA/1 mice, 8 weeks old, 0.30 mL of 3.5% chloral hydrate, 15 mL/kg body weight is recommended).
  b. For the experimental group, IP administration of a single dose of PTZ at a dose of 75 mg/kg, and samples were taken immediately after the onset of S-IRA in the mice.
37. Preparation of blood plasma (Figure 3).
  a. Fully moisten the needle with heparin to prevent blood clotting.
  b. Inject the needle from the apex of the heart and draw blood as slowly as possible to keep the needle free of air.
  c. When transferring blood from the syringe to the centrifuge tube, avoid pushing the needle rapidly and allow the blood to drip slowly into the tube to minimize hemolysis.
  d. After standing for 2 h, centrifuge the samples for 15 min (1000 rpm) to obtain 0.75–0.1 mL plasma.

38. Preparation of heart samples (Figure 4).
  a. After blood collection, perfuse the heart with cold PBS and immediately take the heart sample.
  b. Remove the connective tissue from the surface of the heart, and wash it three times in PBS on ice until the PBS becomes clear.
  c. Use filter paper to absorb the surface and cavity fluid, and then weigh it.
  d. Add a certain amount of PBS, for example, 0.15 g of heart is added with 0.15 × 5 mL, which is 650 μL PBS.
  e. Use an ophthalmic scissors to crush the heart tissue, then whip the solution with an ultrasonic knife until the solution is uniform.
    ***Note:*** This process will generate heat, and the single stirring time should not exceed 4 s. Then place it on ice to cool, and repeat 3–4 times until the homogenate is evenly mixed.
  f. Pre-cool the centrifuge to 4°C and centrifuge the samples for 20 min (12000 rpm).
    ***Note:*** The heart extraction and homogenate preparation process is done on ice.

### ELISA

**Timing: 3 h**

39. Balance the kit at room temperature for 15–30 min before use.

***Note:*** Store the kit in an environment of 2°C–8°C.

40. Take out the required plate from the aluminum foil bag, and set the standard hole, sample hole, blank hole and multiple holes.

***Note:*** If the enzyme label coated plate is not used up after opening, the remaining plate shall be put into a sealed bag and stored away from light.

41. Add 50 μL standard solution of different concentrations to each standard hole. Add 50 μL tested sample with different dilution ratios to sample holes. Blank holes are not added.

**CRITICAL:** It is best to control the addition time within 5 min. If there is a large number of samples, it is recommended to use a row gun for addition.

***Note:*** If the content of the substance to be tested in the sample is too high, dilute it by a certain number of times with the sample diluent. The dilution times for this experiment are 2, 5, and 10 times.

42. Cover the reaction plate with a sealing film and incubate it in a 37°C water bath or incubator for 30 min.

***Note:*** The sealing film is limited to one-time use to avoid cross-contamination.

43. After incubation, peel off the sealing film and discard the liquid.
44. Wash the plate.
  a. Pat the plate dry on absorbent paper.
  b. Wash each hole with 200 μL 1× wash buffer, let stand for 30 s.
  c. Then discard the solution, pat dry on absorbent paper, and repeat washing for 5 times.
45. Add 50 μL horseradish peroxidase labeled detection antibody to the standard holes and sample holes.

***Note:*** The sealing film is limited to one-time use to avoid cross-contamination.

46. Incubate it in a 37°C water bath for 30 min, then discard the solution and repeat washing for 5 times.
47. Before adding the substrate, thoroughly pat the reaction plate dry on clean, chip paper.
48. Color reaction.
  a. Add 50 μL substrate A and 50 μL substrate B to each hole.
  b. Cover the reaction plate with a sealing film.
  c. Place it in the 37°C incubator, incubating it in dark for 15 min.

***Note:*** After adding the chromogenic agent, avoid light in the following procedure.

49. To stop the reaction, add 50 μL termination solution to each hole.
50. Read the absorbance (OD value) on the enzyme marker within 15 min.

***Note:*** Measure the absorbance at 450 nm and 630 nm (background) respectively using Enzyme Labeling Instrument (Thermo Scientific 5250040) (Figures 3 and 4).

**CRITICAL:** The final result is OD value(at 450 nm) minus OD value(at 690 nm) and takes the average with the corresponding multiple holes.

### ECG recording

**Figure 5:**
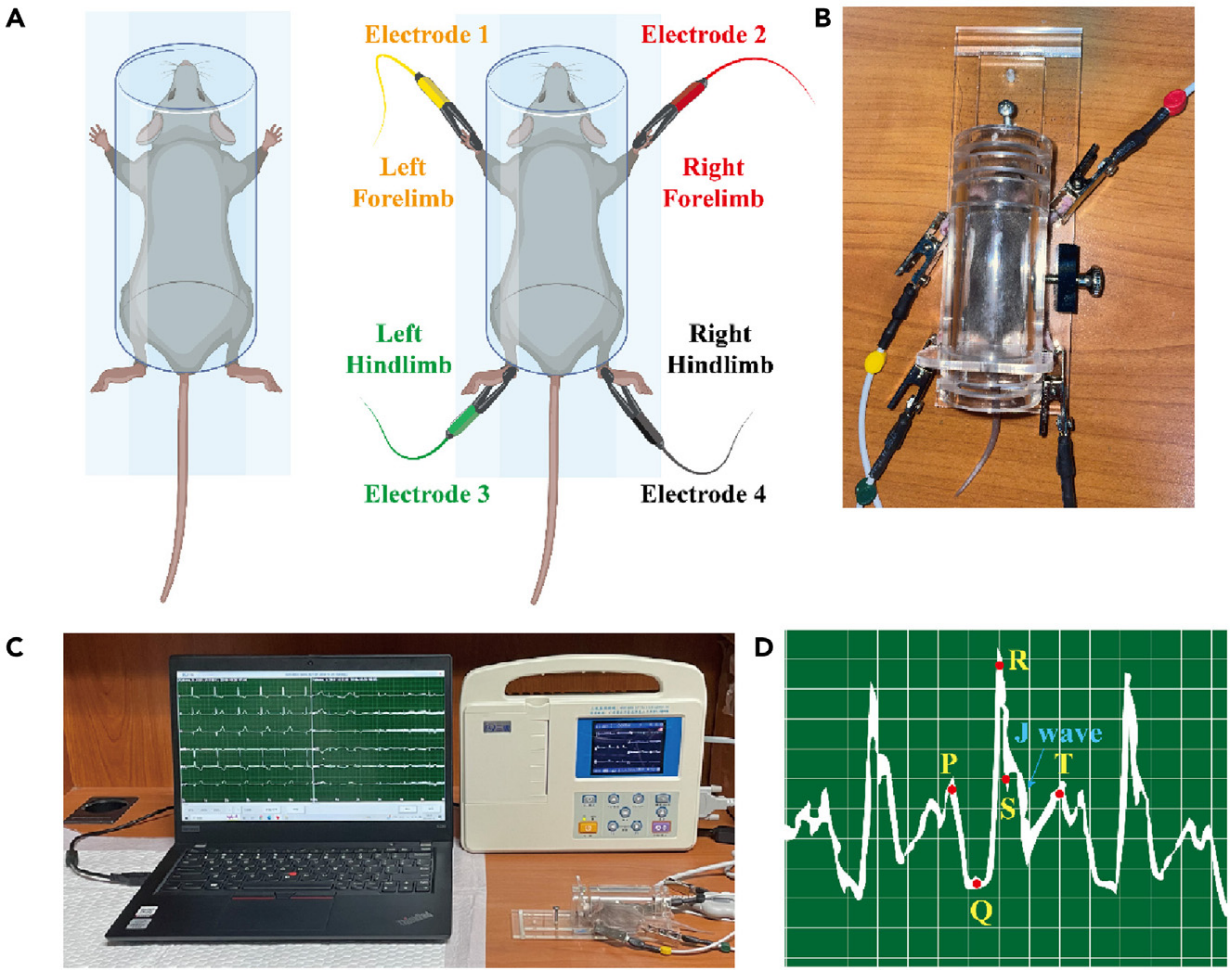
Key procedures for ECG recording and the ECG characteristics of the DBA/1 mice (A) Place the mouse in a plexiglass chamber with a body restraint, and connect the clips of the electrocardiographic lead wires to the mouse limbs in sequence (Right forelimb: red; left forelimb: yellow; right hindlimb: black; left hindlimb: green). (B) Representative image of the DBA/1 mice with clips of electrocardiographic lead wires in place. (C) Representative image of recording ECG and collecting data in the DBA/1 mice. The parameters of the ECG recording are set as follows: the paper speed of ECG was 25 mm/s, and the standard voltage was 1 mV. (D) Electrocardiographic characteristics of the DBA/1 mice. ECG, Electrocardiogram.

51. Connect the machine in advance, including the monitor, limb leads, and software (Figure 5C).

**Figure 6:**
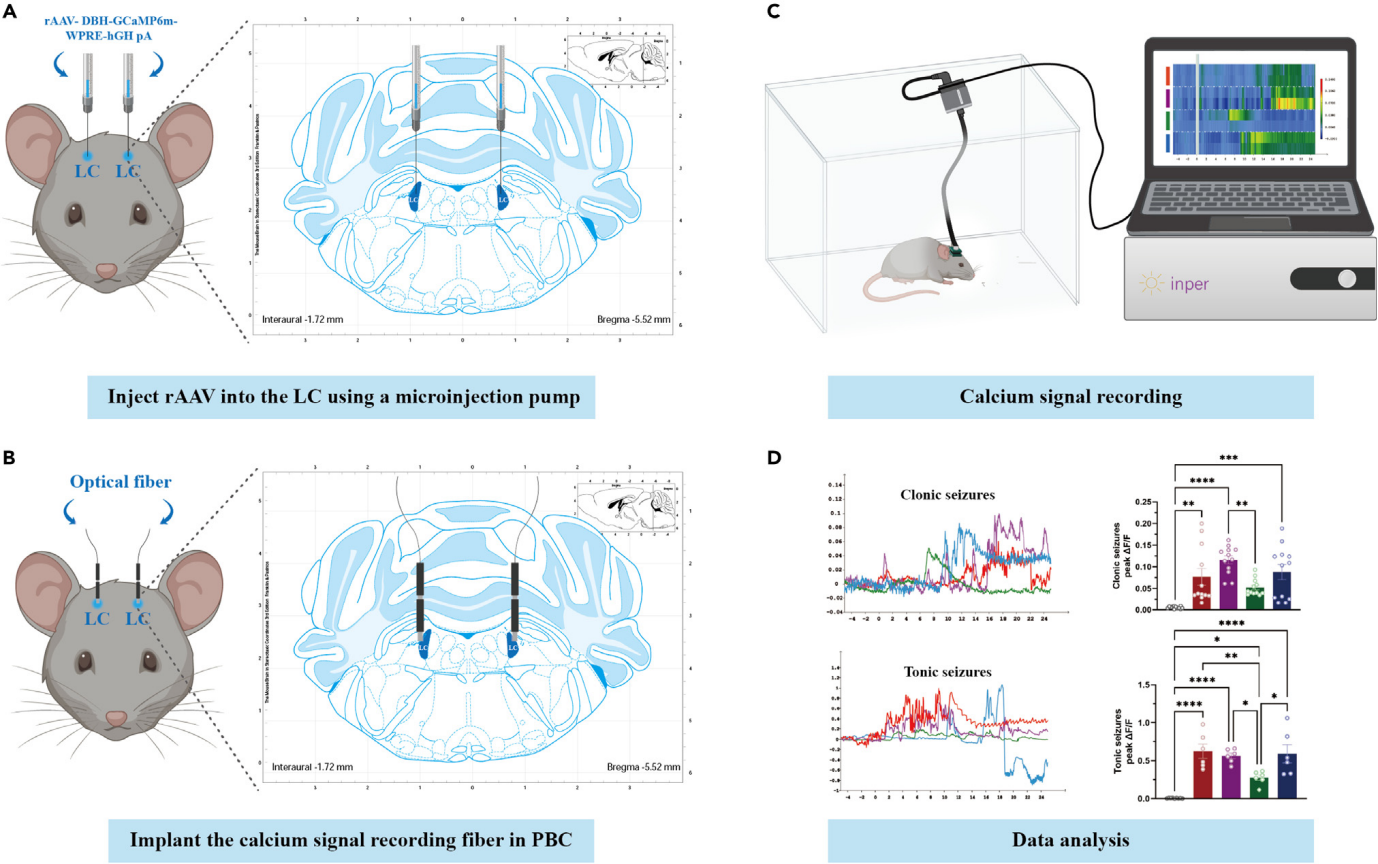
Protocol for the implantation of optical fibers and recording of calcium signals in bilateral LC (A) The location of the LC is determined according to the 4th edition of the mouse brain atlas and microinject (100 nL, at a rate of 40 nL/min) rAAV-DBH-GCaMP6m-WPRE-hGH pA at the stereotaxic coordinates of LC (AP-5.45 mm, ML±0.9 mm, DV-3.65 mm). (B) At the same coordinates, the optical fibers are implanted above LC for 0.10 mm (AP-5.45 mm, ML±0.9 mm, DV-3.65 mm). (C) Schematic illustration of calcium signal recording in DBA/1 mice infected with GCaMP6f in the bilateral LC. (D) The calcium signal data obtained were analyzed to show the activity of neurons in the LC and then perform the statistical analysis between the vehicle and experimental groups. LC, locus coeruleus.
52. Disinfect the limb leads with alcohol before connecting them to increase the conductivity of the limb leads.
53. For ECG recording, place the mouse in a plexiglass chamber with body restraint, and attach the clips of the electrocardiographic lead wires to the limbs of the mouse in sequence.

***Note:*** Connect the electrocardiographic lead wires in the right order (Right forelimb: red; left forelimb: yellow; right hindlimb: black; left hindlimb: green). When connecting the limb leads, be careful not to pull the limbs of the mice too hard to avoid irreversible damage to the limbs of the mice (Figure 5A).

54. After connecting the ECG, wait the mice calm for 5 min before ECG recording.
55. Perform the ECG recording once the mice had adapted to their situation (ECG-2303B, Guangzhou 3Ray Electronics Co., Ltd).
56. For DBA/1 mice in the different pre-treatment groups, ECG was connected at different times.
57. In the acoustic stimulation model, when S-IRA occurred during acoustic stimulation, immediately remove the mice from the isolation box and quickly connect limb leads.

***Note:*** The limb leads of the acoustic stimulation model DBA/1 mice must be connected quickly and timely, requiring two individuals if necessary, otherwise the ECG changes of mice with S-IRA could not be recorded.

58. In the PTZ model, after IP administration of PTZ, tape the mice into a plexiglass chamber with body restraint immediately and record the changes of ECG during the whole course of seizures.
59. For groups that received esmolol injection, perform ECG recording 10 min prior to IP injection of atomoxetine (15 mg/kg) and 10 min after acoustic stimulation or 1 h after IP injection of PTZ (75 mg/kg).
60. Perform ECG recording at a speed of 25 mm/s and sensitivity of 10 mm/mV. Carry out arrhythmia analysis based on ECG features in humans and mice, as described previously.^7^^,^^8^^,^^9^

***Note:*** Define three or more consecutive episodes of spontaneous ventricular electrical depolarizing activity as ventricular tachycardia (VT). Define the premature onset of the QRS complex (wide malformation) with no P wave before it as the ventricular premature beat (VPB). Define slower conduction velocity and longer P-R interval as I atrioventricular block (I AVB). The P-R interval is gradually prolonged until the P wave could not propagate down and the QRS complex disappears, or the P-R interval is stable and the P wave could not be propagated down, which is defined as II atrioventricular block (II AVB). Define the separation of the P wave from the QRS complex as III atrioventricular block (III AVB).

61. The paper output speed of ECG was 25 mm/s, the transverse interval was 0.04 s for each small grid, 0.2 s for each large grid, and the standard voltage was 1 mV (Figure 5).

## Expected outcomes

The atomoxetine-mediated suppression of S-IRA can be significantly reversed by the degeneration of TH^+^ neurons in the LC caused by DSP-4, a selective neurotoxin for the LC noradrenergic system (n = 8). The microinjection of DSP-4 into the LC can significantly reverse the atomoxetine-mediated suppression of S-IRA as well (n = 9). Thus, we found that atomoxetine produced the suppression of S-IRA by targeting the LC in our models.

**Figure 7:**
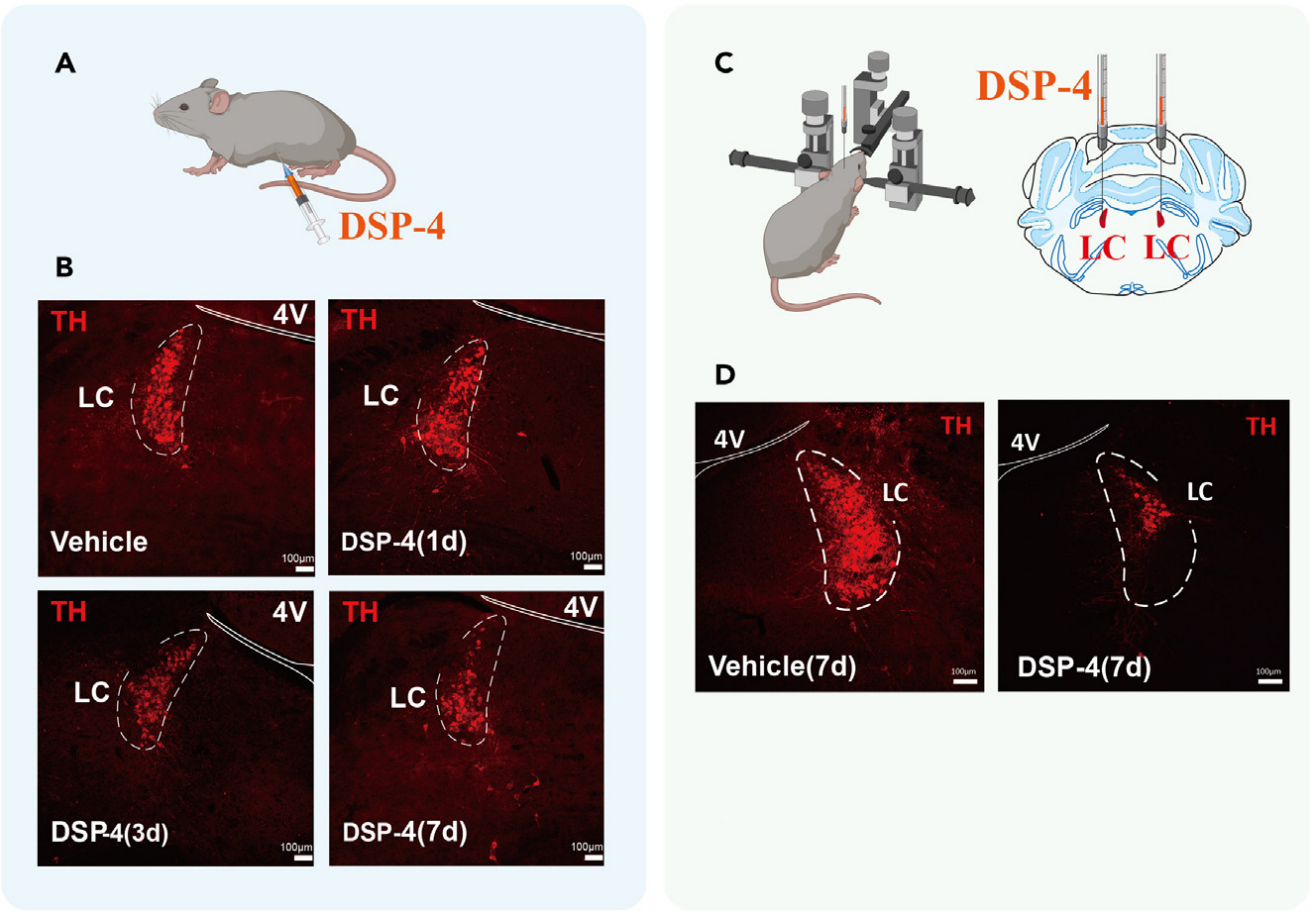
The number of LC^NE^ neurons was significantly reduced after intraperitoneal injection or LC-microinjection of DSP-4 (A) Diagram of the intraperitoneal injection of DSP-4 in DBA/1 mice. (B) Changes of the number of TH^+^ neurons in the LC after intraperitoneal injection of the vehicle or pre-treated with DSP-4 at 1, 3, or 7 days. (C) Diagram of LC-microinjection of DSP-4 in DBA/1 mice. (D) Images of TH + neurons in the LC after microinjection of DSP-4 or vehicle for 7 days. 4V: Fourth ventricle.

We observed the activity of neurons in the LC by recording calcium signalings through optical fibers. The neuronal calcium signaling activity increased in the LC during the clonic seizure phase (n = 6). Virus expression in nerve cells requires at least 3 weeks. The epifluorescence of rAAV-DBH-GCaMP6m-WPRE-hGH Pa is strong enough to observe a green signal without immunohistochemical staining. After the cryostat section, TH + strongly apparent fluorescence and could be observed in the nerve cells of LC (Figure 7). The placement of the optical fiber and cannula tip within the LC a in each mouse is verified by histology. For the figure of histology, please refer to Lian et al. (2023).

As for the changes in the content and activity of TH in the heart, the TH content in both serum from the LV(n = 6) and tissue of the heart (n = 6) is significantly increased following S-IRA, whereas TH activity is significantly decreased after S-IRA in DBA/1 mice (n = 6). As for the changes in the expression of β1-AR in the different regions of the heart, the phosphorylation β1-AR was significantly increased in the compensatory manner of following S-IRA by PTZ (n = 6).

ECG recordings from DBA/1 mice suffering S-IRA evoked by acoustic stimulation and PTZ injection were characterized by a mixture of sinus bradycardia, atrioventricular block, ventricular premature beat, and ventricular tachycardia. Refer to our recently published paper (Lian et al. 2023) for more detailed data and analysis. The incidence of S-IRA of the group pre-treated with atomoxetine and the β1-AR blocker esmolol was significantly greater than the group pre-treated with atomoxetine and vehicle in both the acoustic stimulation model and PTZ model (n = 8). It suggested that esmolol reversed the atomoxetine-mediated suppression of S-IRA by targeting β1-AR on cardiomyocytes.

## Quantification and statistical analysis

All data are presented as the mean ± standard error of the mean (SEM). No statistical methods were used to pre-determine the sample size. GraphPad Prism (GraphPad Software, Inc., San Diego, CA, USA) and SPSS 23 (SPSS Software Inc., Chicago, IL, USA) was used for data display and statistical analysis. The incidence of S-IRA was compared among different groups using the Wilcoxon signed rank test, as these data are nonparametric. The data for seizure scores, latency to AGSZs/GSZs, duration of wild running, clonic seizures, and tonic-clonic seizures were evaluated using one-way analysis of variance (ANOVA) or Kruskal–Wallis H test. One-way ANOVA test or paired-samples T test was used to compare the numbers of TH^+^ cells in the LC among DBA/1 mice. Two-way ANOVA was applied to compare heart rate with and without IP injection of esmolol. One-way ANOVA test was used to compare the clonic seizures and tonic seizures peak ΔF/F. The content and specific enzyme activity of TH were compared between groups using paired-samples T-test. The beta receptor1 and phospho -beta receptor1 content were compared between groups using paired-samples T test as well. Statistical significance was inferred if p < 0.05. ∗p < 0.05, ∗∗p < 0.01, ∗∗∗p < 0.001.

## Limitations

First, we used limb-lead ECG to record the changes in mice during seizures, resulting in unstable ECG due to the struggle of the mice, especially during the initial phase of the measurement. Therefore, ECG recording with implanted leads can be used to avoid the influence of strenuous exercise. Second, since the mice of acoustic stimulation need to be performed in a sound isolation chamber, only the ECG during S-IRA and the death of the mice of acoustic stimulation were recorded in this experiment. We may consider the mouse telemetry implantation surgery using ECG recording devices to monitor ECG changes throughout the whole experiment next time to avoid missing the immediate changes of the ECG and avoid the fluctuation of ECG because of struggle of the mice. Finally, there are limitations with our cardiac activity monitoring. More perfect recordings of heart indexes, such as the ejection fraction, will provide insight into cardiac function during SUDEP.

## Troubleshooting

### Problem 1

At the time of serum collection, the specimen is prone to hemolysis. Substances with peroxidase activity will be released by hemolysis, and hemolyzed samples may increase non-specific color development. It is very important to take care to avoid hemolysis during collection (to step 37 of “ELISA sampling”).

### Potential solution

Before drawing blood, draw a small amount of heparin, turn the syringe to cover the wall of the syringe with heparin, and then expel the heparin from the syringe. Remove the needle immediately after blood collection, and inject the blood slowly into the dry test tube along the tube wall. Be careful not to inject the test tube hard or quickly to avoid the rupture of red blood cells, and do not inject foam into the test tube. After injection into the tube, take care to avoid shaking in order to prevent blood cells from rupture and hemolysis.

### Problem 2

The timing of ECG recording was different between the acoustic stimulation model and the PTZ model. In particular, in the acoustic stimulation model, the trend of EEG changes throughout S-IRA could not be fully recorded if the limb leads were not connected in time (to step 53 of “ECG recording”).

### Potential solution

In the acoustic stimulation model, when S-IRA occurred during acoustic stimulation, immediately remove the mice from the isolation box and quickly connect limb leads and requiring two individuals if necessary. The ECG changes were recorded from the time of connection to the mice until the mice died of S-IRA. In the PTZ model, after IP administration of PTZ, tape the mice into the plexiglass chamber with body restraint immediately and record the changes of ECG during the whole course of seizures for 1 h.

### Problem 3

Whether esmolol, which causes slow heart rate and cardiac conduction block, might also cause death in mice remains unclear and needs to be distinguished from the effect of reversing atomoxetine in reducing S-IRA in mice (to step 55 of “ECG recording”).

### Potential solution

To observe the incidence of death among DBA/1 mice as well as esmolol-induced heart rate changes, the group of mice (n = 6) used to confirm S-IRA 24 h before the start of the experiment was pre-treated with esmolol (50 mg/kg, IP) without administration of atomoxetine and subjected to acoustic stimulation in the same manner. For DBA/1 mice in all of the different pre-treatment groups, ECG was performed before acoustic stimulation and after S-IRA.

### Problem 4

During the development of seizures and the occurrence of S-IRA, the ECG will show different arrhythmias, so it is very important to define the type of arrhythmia (to step 60 of “ECG recording”).

### Potential solution

**Figure 8:**
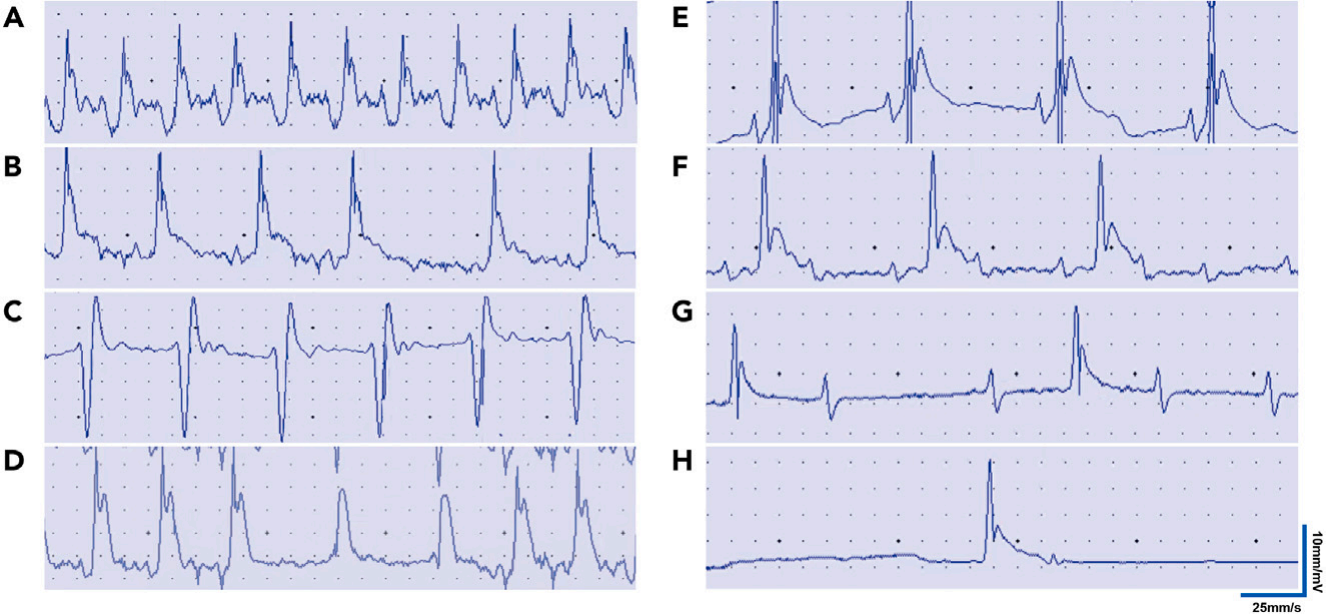
Schematic diagram of arrhythmia ECG in mice (A) Normal ECG in DBA/1 mice. (B) Atrioventricular block can be observed on ECG when mice experience clonic seizures. (C) Typical ECG of Ventricular Tachycardia (VT). (D) Typical ECG of Ventricular Premature Beat (VPB). (E) Typical ECG of I Atrioventricular Block (I AVB). (F) Typical ECG of II Atrioventricular Block (II AVB). (G) Typical ECG of III Atrioventricular Block (III AVB). (H) Typical ECG of Sudden cardiac arrest.

VT: three or more consecutive episodes of spontaneous ventricular electrical depolarizing activity. VPB: premature onset of QRS complex (wide malformation) and no P wave before it. I AVB: slower conduction velocity and longer P-R interval. II AVB: The P-R interval is gradually prolonged until the P wave could not propagate down and the QRS complex disappears, or the P-R interval is stable and the P wave could not be propagated down. III AVB: the separation of the P wave from the QRS complex (Figure 8).

### Problem 5

It is difficult to determine the effective administration time of DSP-4, a selective neurotoxin for targeting the LC noradrenergic system (to step 6 of “reagents preparation”).

### Potential solution

To investigate the effects of DSP-4 on noradrenergic neurons in the LC, DBA/1 mice were pre-treated with DSP-4 (50 mg/kg, IP) or vehicle without administration of atomoxetine and acoustic stimulation. These DBA/1 mice were sacrificed and perfused after 1, 3, or 7 days for counting of the TH^+^ cells to select the best effective administration time. TH^+^ cells were counted in 5 sections for each animal, and the average numbers were compared between the DSP-4 treated and vehicle-treated mice (Figure 7).

## Resource availability

### Lead contact

Further information and requests should be directed to and will be fulfilled by the lead contact, Honghai Zhang (zhanghonghai_0902@163.com).

### Materials availability

This study did not generate new unique reagents.

## Data Availability

This study did not generate new unique data or code.
